# GAVIN: Gene-Aware Variant INterpretation for medical sequencing

**DOI:** 10.1186/s13059-016-1141-7

**Published:** 2017-01-16

**Authors:** K. Joeri van der Velde, Eddy N. de Boer, Cleo C. van Diemen, Birgit Sikkema-Raddatz, Kristin M. Abbott, Alain Knopperts, Lude Franke, Rolf H. Sijmons, Tom J. de Koning, Cisca Wijmenga, Richard J. Sinke, Morris A. Swertz

**Affiliations:** 1 University of Groningen, University Medical Center Groningen, Genomics Coordination Center, Groningen, The Netherlands; 2 Department of Genetics, University of Groningen, University Medical Center Groningen, Groningen, The Netherlands

**Keywords:** Clinical next-generation sequencing, Variant classification, Automated protocol, Gene-specific calibration, Allele frequency, Protein impact, Pathogenicity prediction

## Abstract

We present Gene-Aware Variant INterpretation (GAVIN), a new method that accurately classifies variants for clinical diagnostic purposes. Classifications are based on gene-specific calibrations of allele frequencies from the ExAC database, likely variant impact using SnpEff, and estimated deleteriousness based on CADD scores for >3000 genes. In a benchmark on 18 clinical gene sets, we achieve a sensitivity of 91.4% and a specificity of 76.9%. This accuracy is unmatched by 12 other tools. We provide GAVIN as an online MOLGENIS service to annotate VCF files and as an open source executable for use in bioinformatic pipelines. It can be found at http://molgenis.org/gavin.

**Electronic supplementary material:**

The online version of this article (doi:10.1186/s13059-016-1141-7) contains supplementary material, which is available to authorized users.

## Background

Only a few years ago, the high costs and technological challenges of whole exome and whole-genome sequencing (WGS) were limiting their application. Today, the practice of human genome sequencing has become routine even within the healthcare sector. This is leading to new and daunting challenges for clinical and laboratory geneticists [1]. Interpreting the thousands of variations observed in DNA and determining which are pathogenic and which are benign is still difficult and time-consuming, even when variants are prioritized by state-of-the-art in silico prediction tools and heuristic filters [2]. Using the current, largely manual, variant classification protocols, it is not feasible to assess the thousands of genomes per year now produced in a single hospital. It is the challenge of variant assessment which now impedes the effective uptake of next-generation sequencing (NGS) into routine medical practice.

The recently introduced CADD [3] scores are a promising alternative [4]. These are calculated on the output of multiple in silico tools in combination with other genomic features. They trained a computer model on variants that have either been under long-term selective evolutionary pressure or none at all. The result was an estimation of deleteriousness for variants in the human genome, whether already observed or not. It has been shown to be a strong and versatile predictor for pathogenicity [3] with applications and popular uptake in many areas of genome research. Variant interpretation in a diagnostic setting may also benefit from this method. However, successful uptake requires a translational effort because CADD scores are intended to rank variants, whereas NGS diagnostics requires a discrete classification for each variant. For example, SIFT [5] probabilities are used to partition “tolerated” (probability >0.05) from “damaging” variants (probability < =0.05). CADD scores may be used to define such a binary classifier, but using a single, arbitrary cutoff value is not recommended by the CADD authors [6]. Moreover, clinicians and laboratories cannot rely on a single threshold approach because it has been shown that individual genes differ in their cutoff thresholds for what should be considered the optimal boundary between pathogenic or benign [4]. This issue has been partly addressed by mutation significance cutoff (MSC) [7], which provides gene-based CADD cutoff values to remove inconsequential variants safely from sequencing data. While MSC aims to quickly and reliably reduce the number of benign variants left to interpret, it was not developed to detect/classify pathogenic variants.

The challenge is thus to find robust algorithms that classify both pathogenic and benign variants accurately and that fit into existing best practice, diagnostic filtering protocols [8]. Implementing such tools is not trivial because genes have different levels of tolerance to various classes of variants that may be considered harmful [9]. In addition, the pathogenicity estimates for benign variants are intrinsically lower because these are more common and of less severe consequence on protein transcription. Comparing the prediction score distributions of pathogenic variants with those of typical benign variants is therefore biased and questionable. Using such an approach means it will be unclear how well a predictor truly performs if a benign variant shares the same allele frequency and consequence with known pathogenic variants. Here, we present Gene-Aware Variant Interpretation (GAVIN), a new method that addresses these issues by gene-specific calibrations on closely matched sets of variants. GAVIN delivers accurate and reliable automated classification of variants for clinical application.

## Results

### Development of GAVIN

GAVIN classifies variants as benign, pathogenic, or a variant of uncertain significance (VUS). It considers ExAC [9] minor allele frequency, SnpEff [10] impact, and CADD score using gene-specific thresholds. For each gene, we ascertained ExAC allele frequencies and effect impact distributions of variants described in ClinVar (November 2015 release) [11] as pathogenic or likely pathogenic. From the same genes, we selected ExAC variants that were not present in ClinVar as a benign reference set. We stratified this benign set to match the pathogenic set with respect to the effect impact distribution and minor allele frequencies (MAFs). Using these comparable variant sets we calculated gene-specific mean values for CADD scores (across all genes, the pathogenic mean of means was 28.44 and that of benign was 23.08) and MAFs, as well as 95th percentile sensitivity/specificity CADD thresholds for both benign and pathogenic variants. Of 3237 genes that underwent the calibration process, we found 2525 informative gene calibrations, i.e. thresholds for CADD, effect impact, pathogenic 95th percentile MAFs, or a combination thereof (see Additional file 1: Table S1). We used fixed genome-wide classification thresholds as a fallback strategy based on CADD scores <15 for benign, >15 for pathogenic, and on a MAF threshold of 0.00426, which was the mean of all gene-specific pathogenic 95th percentile MAFs. This allowed classification when insufficient variant training data were available to allow for gene-specific calibrations or when the gene-specific rules failed to classify a variant. Based on the gene calibrations we then implemented GAVIN, which can be used online or via commandline (see http://molgenis.org/gavin) to perform variant classification.

### Performance benchmark

To test the robustness of GAVIN, we evaluated its performance using six benchmark variant classification sets from VariBench [12], MutationTaster2 [13], ClinVar (only recently added variants that were not used for calibrating GAVIN), and a high-quality variant classification list from the University Medical Center Groningen (UMCG) genome diagnostics laboratory. These sets and the origins of their variants and classifications are described in Table 1. The combined set comprises 25,765 variants (17,063 benign, 8702 pathogenic). All variants were annotated by SnpEff, ExAC, and CADD prior to classification by GAVIN. To assess the clinical relevance of our method, we stratified the combined set into clinically relevant variant subsets based on organ-system specific genes. We formed 18 subset panels such as Cardiovascular, Dermatologic, and Oncologic based on the gene-associated physical manifestation categories from Clinical Genomics Database [14]. A total of 11,679 out of 25,765 variants were not linked to clinically characterized genes and formed a separate panel (see Table 2 for an overview, which includes the number of pathogenic variants in each panel). In addition, we assessed the performance of GAVIN in compared to 12 common in silico tools for pathogenicity prediction: MSC (using two different settings), CADD (using three different thresholds), SIFT [5], PolyPhen2 [15], PROVEAN [16], Condel [17], PON-P2 [18], PredictSNP2 [19], FATHMM-MKL [20], GWAVA [21], FunSeq [22], and DANN [23].

**Table 1:** Variant and classification origins of the benchmark datasets used

Dataset	Benign variants (n)	Pathogenic variants (n)	Origin
VariBench tolerance DS7, training set	11,347	6143	PhenCode database, IDbases, and 18 individual LSDBs
VariBench tolerance DS7, test set	1377	510	PhenCode database, IDbases, and 18 individual LSDBs
MutationTaster2 benchmark set	1194	161	HGMD Professional and 1000 Genomes
ClinVar (additions of Nov 2015 to Feb 2016)	1668	1688	Submissions by clinical molecular geneticists, expert panels, diagnostic laboratories, and companies
UMCG, variants exported from clinical diagnostic interpretation software	1176	174	Clinical diagnostic classifications of variants in cardiology, dermatology, epilepsy, dystonia, and preconception screening
UMCG, germline variants for familial cancer cases	301	26	Hereditary cancer variant classifications by an MD following ACMG guidelines
Total	17,063	8702	25,765

**Table 2:** Stratification of the combined variant dataset into manifestation categories

CGD manifestation panel	Genes (n)	Variants (n)	Likely pathogenic/pathogenic variants (n)
Allergy/Immunology/Infectious	253	1952	1324
Audiologic/Otolaryngologic	217	1215	668
Biochemical	354	2538	1933
Cardiovascular	446	4360	2408
Craniofacial	387	1861	1106
Dental	80	783	518
Dermatologic	345	2749	1662
Endocrine	240	1801	1340
Gastrointestinal	338	2351	1620
Genitourinary	149	1026	753
Hematologic	267	2571	1914
Musculoskeletal	676	4935	2864
Neurologic	1012	6363	4055
Obstetric	34	223	140
Oncologic	203	2157	1207
Ophthalmologic	479	3649	2406
Pulmonary	90	717	485
Renal	302	2143	1459
*NotInCGD*	*5806*	*11,679*	*122*

The categories are defined by Clinical Genomics Database and are associated to clinically relevant genes. Variants were allocated to the manifestation categories based on their gene and were placed in multiple categories if a gene was associated to multiple manifestations

**Fig. 1:**
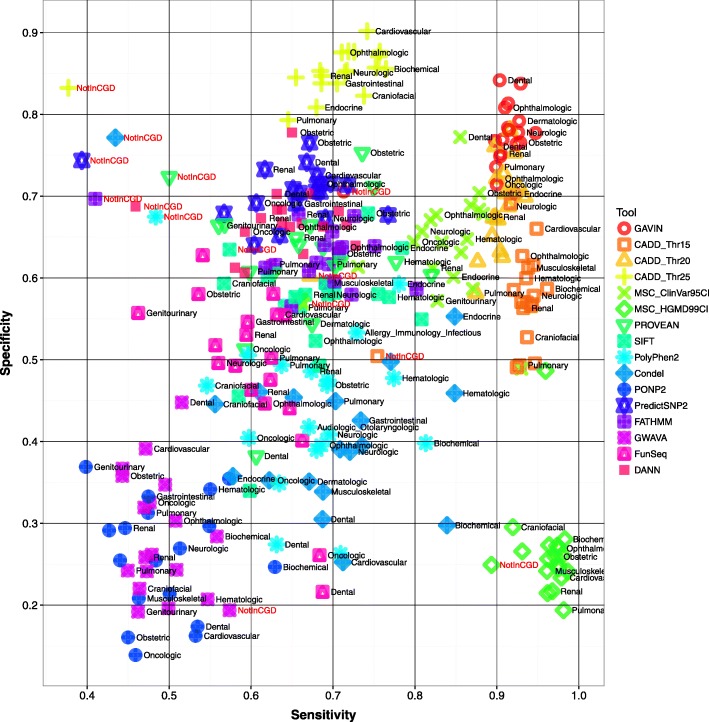
Performance of GAVIN and other tools across different clinical gene sets. Prediction quality is measured as sensitivity and specificity, i.e. the fraction of pathogenic variants correctly identified and the fraction of misclassifications/non-classifications while doing so

Across all test sets, GAVIN achieved a median sensitivity of 91.4% and a median specificity of 76.9%. Other tools with >90% sensitivity were CADD (93.6% at threshold 15, with specificity 57.1%, and 90.4% at threshold 20, with specificity 68.8%) and MSC (97.1%, specificity 25.7%). The only tool with a higher specificity was CADD at threshold 25 (85.3%, sensitivity 71.5%). See Table 3 for an overview of tool performance or Fig. 1 for more detail. In all the clinical gene sets GAVIN scored >89.7% sensitivity, including >92% for Cardiovascular, Biochemical, Obstetric, Neurologic, Hematologic, Endocrine, and Dermatologic genes. The non-clinical genes scored 71.3%. The specificity in clinical subsets ranged from 70.3% for Endocrine to 84.2% for Dental. Non-clinical gene variants were predicted at 70.6% specificity. See Additional file 2: Table S2 for detailed results.

**Table 3:** Performance overview of all tested tools

Tool	Median sensitivity (%)	Median specificity (%)
CADD (thr. 15)	93.6	57.1
CADD (thr. 20)	90.4	68.8
CADD (thr. 25)	71.5	85.3
Condel	70.3	39.5
DANN	63.8	66.7
FATHMM	69.5	61.9
FunSeq	61.7	50.2
GAVIN	91.4	76.9
GWAVA	47.6	26.2
MSC_ClinVar95CI	84.7	64.4
MSC_HGMD99CI	97.1	25.7
PolyPhen2	68.0	46.8
PONP2	47.5	26.9
PredictSNP2	66.8	70.6
PROVEAN	65.9	62.1
SIFT	67.9	57.9

### Added value of gene-specific calibration

We then investigated the added value of using gene-specific thresholds on classification performance relative to using genome-wide thresholds. We bootstrapped the performance on 10,000 random samples of 100 benign and 100 pathogenic variants. These variants were drawn from the three groups of genes described in “Methods”: (1) genes for which CADD was significantly predictive for pathogenicity (*n* = 681); (2) genes where CADD was not significantly predictive (*n* = 732); and (3) genes with scarce variant data available for calibration (*n* = 774). For each of these sets we compared the use of gene-specific CADD and MAF classification thresholds with that of genome-wide filtering rules.

**Fig. 2:**
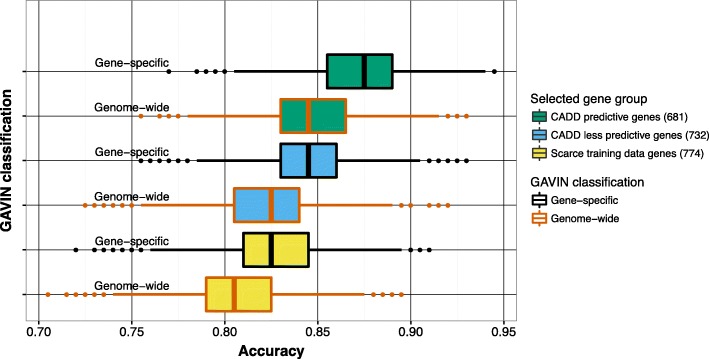
Comparison of gene-specific classification thresholds with genome-wide fixed thresholds in three groups of genes: 737 genes for which CADD is predictive, 684 genes for which CADD is less predictive, and 766 genes with scarce training data. For each group, 10,000 sets of 100 benign and 100 pathogenic variants were randomly sampled and tested from the full set of 25,765 variants and accuracy was calculated for gene-specific and genome-wide CADD and MAF thresholds

We observed the highest accuracy on genes for which CADD had significant predictive value and for the gene-specific classification method (median accuracy = 87.5%); this was significantly higher than using the genome-wide method for these same genes (median accuracy = 84.5%, Mann–Whitney U test *p* value <2.2e-16). For genes for which CADD had less predictive value, we found a lower overall performance, but still reached a significantly better result using the gene-specific approach (median accuracy = 84.5% versus genome-wide 82.5%, *p* value <2.2e-16). Lastly, the worst performance was seen for variants in genes with scarce training data available. The gene-specific performance, however, was still significantly better than using genome-wide thresholds (median accuracy = 82.5% and 80.5%, respectively, *p* value = 2.2e-16). See Fig. 2.

## Discussion

We have developed GAVIN, a method for automated variant classification using gene-specific calibration of classification thresholds for benign and pathogenic variants.

Our results show that GAVIN is a powerful classifier with consistently high performance in clinically relevant genes. The robustness of our method arises from a calibration strategy that first corrects for calibration bias between benign and pathogenic variants, in terms of consequence and rarity, before calculating the classification thresholds. A comprehensive benchmark demonstrates a unique combination of high sensitivity (>90%) and high specificity (>70%) for variants in genes related to different organ systems. This is a significant improvement over existing tools that tend to achieve either a high sensitivity (MSC, CADD at lower thresholds) or a high specificity (PredictSNP2, CADD at higher thresholds). A high sensitivity is crucial for clinical interpretation because pathogenic variants should not be falsely discarded. In addition, having a higher specificity means that the results will be far less “polluted” with false positives and thus less risk of patients being given a wrong molecular diagnosis. GAVIN decreases false positives by 10–20% compared to using CADD for the same purpose, thereby reducing interpretation time. The difference between using a high and low performance method can be dramatic in practice. In a hypothetical example, GAVIN would make downstream variant interpretation twice as effective as a low performance method, with more sensitive detection of pathogenic variants (see Additional file 3: Table S3).

Even though an optimal combination of sensitivity and specificity may be favorable in general terms, there may still be a need for tools that perform differently. The MSC gene-specific thresholds based on HGMD [24] at 99% confidence interval show a very high sensitivity (97.1%), but at the expense of a very low specificity (25.7%). Such low specificity thresholds will pick up almost all the pathogenic variants with scores exceeding gene thresholds. This allows safe removal (<3% error) of benign variants that fall below these thresholds, which was their authors’ aim. However, this tool cannot detect pathogenic variants due to its low specificity. Other tools, such as PON-P2, may show a relatively low performance, but not necessarily because of true errors. Such tools may simply be very “picky” and only return a classification when the verdict carries high confidence. If we ignore the variants that PON-P2 did not classify (52% of total benchmark variants) and only consider how many of the variants that it did classify were correct, we find a positive predictive value of 96% and a negative predictive value of 94%. Thus, while this tool might not be useful for exome screening because too many pathogenic variants would be lost, it can still be an excellent choice for further investigation of interesting variants. We would therefore emphasize that appropriate tools should be selected depending on the question or analysis protocol used and by taking their strengths and weaknesses into account.

Not surprisingly, we could confirm that the use of gene-specific thresholds instead of genome-wide thresholds led to a consistent and significant improvement of classification performance. This shows the added value of our strategy. Overall performance was slightly lower in genes for which CADD has limited predictive value and even lower in genes with few “gold standard” pathogenicity data available. Evaluating variants in uncharacterized genes is rare in clinical diagnostics, although it may occur when exome sequencing is aimed at solving complex phenotypes or undiagnosed cases. Nevertheless, GAVIN is likely to improve continuously in an increasing number of genes, propelled by the speed at which pathogenic variants are now being reported. The results of this paper are based on the ClinVar release of November 2015 and comprise 2525 informative gene calibrations, i.e. thresholds for CADD, impact, MAF, or a combination thereof. When we calibrate on the September 2016 ClinVar release, we obtain more informative gene calibrations (2770) with stable gene CADD thresholds (mean pathogenic difference of 0.1%, mean benign difference of 1.1%) and a slight drop in pathogenic MAF (0.00426 to 0.00346). Using these newer calibrations, the benchmark performance of GAVIN increases to 91.7% sensitivity (up from 91.4%) and 78.2% specificity (up from 76.9%). If this trend continues and (2770-2525)/10 = 24.5 genes per month are added, we estimate that calibrating all disease genes in CGD (3316 per Sept. 2016) will take another (3316-2770)/24.5/12 = 1.86 ≈ 2 years.

With GAVIN, we were also able to demonstrate the residual power of CADD scores as a predictor for pathogenicity on a gene-by-gene basis, revealing that the scores are informative for many genes (these results can be accessed at http://molgenis.org/gavin). There are several possible explanations for potential non-informativity of CADD scores. It may have bias towards the in silico tools and sources it was trained on, limiting their predictiveness for certain genomic regions or disease mechanisms [25]. Furthermore, calibration of pathogenic variants could be difficult in genes with high damage tolerance, i.e. having many missense or loss-of-function mutations [26]. In addition, calibration may be impaired by false input signals, such as an incorrect pathogenic classification in ClinVar or inclusion of disease cohorts in large databases such as ExAC could misrepresent allele frequencies [27]. Lastly, pathogenic variants could have a low penetrance or their effect mitigated by genetic modifiers, causing high deleteriousness to be tolerated in the general population against expectations [28].

The field of clinical genomics is now moving towards interpretation of non-coding disease variants (NCVs) identified by WGS [29]. A number of recently introduced metrics, including EIGEN [30], FATHMM-MKL, DeepSEA [31], and GWAVA, specialize in predicting the functional effects of non-coding sequence variation. When a pathogenic NCV reference set of reasonable quantity becomes available, a calibration strategy as described here will be essential to be able to use these metrics effectively in whole-genome diagnostics.

## Conclusions

GAVIN provides an automated decision-support protocol for classifying variants, which will continue to improve in scope and precision as more data are publicly shared by genome diagnostic laboratories. Our approach bridges the gap between estimates of genome-wide and population-wide variant pathogenicity and contributes to their practical usefulness for interpreting clinical variants in specific patient populations. Databases such as ClinVar contain a wealth of implicit rules now used manually by human experts to classify variants. Rules on minor allele frequencies, estimated effect impact, and CADD scores are deduced and employed by GAVIN to classify variants that have not been seen before.

We envision GAVIN accelerating NGS diagnostics and becoming particularly beneficial as a powerful (clinical) exome screening tool. It can be used to quickly and effectively detect over 90% of pathogenic variants in a given dataset and to present these results with an unprecedented small number of false positives. It may especially serve laboratories that lack the resources necessary to perform reliable and large-scale manual variant interpretation for their patients and spur the development of more advanced gene-specific classification methods. We provide GAVIN as an online MOLGENIS [32] web service to browse gene calibration results and annotate VCF files and as a commandline executable including open source code for use in bioinformatic pipelines. GAVIN can be found at http://molgenis.org/gavin.

## Methods

### Calibration of gene-specific thresholds

We downloaded ClinVar (variant_summary.txt.gz from ClinVar FTP, last modified date: 05/11/15) and selected GRCh37 variants that contained the word “pathogenic” in their clinical significance. These variants were matched against the ClinVar VCF release (clinvar.vcf.gz, last modified date: 01/10/15) using RS (Reference SNP) identifiers in order to resolve missing indel notations. On the resulting VCF, we ran SnpEff version 4.1 L with these settings: hg19 -noStats -noLog -lof -canon -ud 0. As a benign reference set, we selected variants from ExAC (release 0.3, all sites) from the same genic regions with +/– 100 bases of padding on each side to capture more variants residing on the same exon. We first determined the thresholds for gene-specific pathogenic allele frequency by taking the ExAC allele frequency of each pathogenic variant or assigning zero if the variant was not present in ExAC, and calculating the 95th percentile value per gene using the R7 method from Apache Commons Math version 3.5. We filtered the set of benign variants with this threshold to retain only variants that were rare enough to fall into the pathogenic frequency range.

Following this step, the pathogenic impact distribution was calculated as the relative proportion of the generalized effect impact categories, as annotated by SnpEff on the pathogenic variants. The same calculation was performed on the benign variants uniquely present in ExaC. To facilitate this, we annotated ExAC with SnpEff (4.1 L, same settings as above) to get the same impact, transcript, and gene nomenclature as our ClinVar set. Overlapping genes were not an issue because SnpEff variant annotations include the gene symbol to which an estimated impact is applicable and subsequently only those matching impacts were considered. The benign variants were subsequently downsized to match the impact distribution of the pathogenic variants.

For instance, in the case of 407 pathogenic MYH7 variants, we found a pathogenic allele frequency threshold of 4.942e-5, and an impact distribution of 5.41% HIGH, 77.4% MODERATE, 17.2% LOW, and 0% MODIFIER. We defined a matching set of benign variants by retrieving 1799 MYH7 variants from ExAC (impact distribution: 2% HIGH, 23.59% MODERATE, 32.59% LOW, 41.82% MODIFIER), from which we excluded known ClinVar pathogenic variants (*n* = 99), variants above the AF threshold (*n* = 246), and removed interspersed variants using a non-random “step over” algorithm until the impact distribution was equalized (*n* = 960). We thus reached an equalized benign set of 494 variants, having an impact distribution of 5.47% HIGH, 77.33% MODERATE, 17.21% LOW, and 0% MODIFIER).

We then obtained the CADD scores for all variants and tested whether there was a significant difference in scores between the sets of pathogenic and benign variants for each gene, using a Mann–Whitney U test. Per gene we determined the mean CADD score for each group and also the 95th percentile sensitivity threshold (detection of most pathogenic variants while accepting false positives) and 95th percentile specificity threshold (detection of most benign variants while accepting false negatives) using the Percentile R7 function. All statistics were done with Apache Commons Math version 3.5. This calibration process was repeated for 3237 genes, resulting in 2525 genes for which we learned classification rules involving pathogenic variant MAF, effect impact distribution, CADD score thresholds, or a combination thereof.

On average, CADD scores were informative of pathogenicity. The mean benign variant CADD score across all genes was 23.08, while the mean pathogenic variant CADD score was 28.44, a mean difference of 5.36 (σ = 4.80). Of 3237 genes that underwent the calibration process, we found 681 “CADD predictive” genes that had a significantly higher CADD score for pathogenic variants than for benign variants (Mann–Whitney U test, *p* value <0.05). Interestingly, we also found 732 “CADD less predictive” genes, for which there was no proven difference between benign and pathogenic variants (*p* value >0.05 despite having ≥5 pathogenic and ≥5 benign variants in the gene). For 774 genes, there were few calibration data available (<5 pathogenic or <5 benign variants), resulting in no significant difference (*p* value >0.05) between CADD scores of pathogenic and benign variants. We also found 159 genes for which effect impact alone was predictive, meaning that a certain impact category was unique for pathogenic variants compared to benign variants. For instance, if we observe HIGH impact pathogenic variants (frame shift, stopgain, etc.) for a given gene, whereas benign variants only reach MODERATE impact (missense, inframe insertion, etc.), we use this criterion as a direct classifier. No further CADD calibration was performed on these genes. In summary, the total set of 3237 genes comprises 681 “CADD predictive” genes + 732 “CADD less predictive” genes + 774 “little calibration data” genes + 159 “impact predictive” + 178 genes with only pathogenic MAF calibrated + 712 genes without calibration due to less than 2 ClinVar or ExAC variants available + 1 artifact where population CADD was greater than pathogenic CADD. See Additional file 1: Table S1 for details.

### Variant sets for benchmarking

We obtained six variant sets that had been classified by human experts. These datasets were used to benchmark the in silico variant pathogenicity prediction tools mentioned in this paper. Variants from the original sets may sometimes be lost due to conversion of cDNA/HGVS notation to VCF.

The VariBench protein tolerance dataset 7 (http://structure.bmc.lu.se/VariBench/) contains disease-causing missense variations from the PhenCode [33] database, IDbases [34], and 18 individual LSDBs [12]. The training set we used contained 17,490 variants, of which 11,347 were benign and 6143 pathogenic. The test set contained 1887 variants, of which 1377 were benign and 510 pathogenic. We used both the training set and test set as benchmarking sets.

The MutationTaster2 [13] test set contains known disease mutations from HGMD [24] Professional and putatively harmless polymorphisms from 1000 Genomes. It is available at http://www.mutationtaster.org/info/Comparison_20130328_with_results_ClinVar.html. This set contains 1355 variants, of which 1194 are benign and 161 pathogenic.

We selected 1688 pathogenic variants from ClinVar that were added between November 2015 and February 2016 as an additional benchmarking set, since our method was based on the November 2015 release of ClinVar. We supplemented this set with a random selection of 1668 benign variants from ClinVar, yielding a total of 3356 variants.

We obtained an in-house list of 2359 variants that had been classified by molecular and clinical geneticists at the University Medical Center Groningen. These variants belong to patients seen in the context of various disorders: cardiomyopathies, epilepsy, dystonia, preconception carrier screening, and dermatology. Variants were analyzed according to Dutch medical center guidelines [35] for variant interpretation, using Cartagenia Bench Lab™ (Agilent Technologies) and Alamut® software (Interactive Biosoftware) by evaluating in-house databases, known population databases (1000G [36], ExAC, ESP6500 at http://evs.gs.washington.edu/EVS/, GoNL [37]), functional effect, and literature searches. Any ClinVar variants included in the November 2015 release were removed from this set to prevent circular reasoning, resulting in a total of 1512 variants, with 1176 benign/likely benign (merged as Benign), 162 VUS, and 174 pathogenic/likely pathogenic (merged as Pathogenic).

From the UMCG diagnostics laboratory we also obtained a list of 607 variants seen in the context of familial cancers. These were interpreted by a medical doctor according to ACMG guidelines [8]. We removed any ClinVar variants (November 2015 release), resulting in 395 variants, with 301 benign/likely benign (merged as Benign), 68 VUS, and 26 likely pathogenic/pathogenic (merged as Pathogenic).

### Variant data processing and preparation

We used Ensembl VEP (http://grch37.ensembl.org/Homo_sapiens/Tools/VEP/) to convert cDNA/HGVS notations to VCF format. Newly introduced N-notated reference bases were replaced with the appropriate GRCh37 base, and alleles were trimmed where needed (e.g. “TA/TTA” to “T/TT”). We annotated with SnpEff (version 4.2) using the following settings: hg19 -noStats -noLog -lof -canon -ud 0. CADD scores (version 1.3) were added by running the variants through the CADD webservice (available at http://cadd.gs.washington.edu/score). ExAC (release 0.3) allele frequencies were added with MOLGENIS annotator (release 1.16.2). We also merged all benchmarking sets into a combined file with 25,995 variants (of which 25,765 classified as benign, likely benign, likely pathogenic, or pathogenic) for submission to various online in silico prediction tools.

### Execution of in silico predictors

The combined set of 25,765 variants was classified by the in silico variant pathogenicity predictors (MSC, CADD, SIFT, PolyPhen2, PROVEAN, Condel, PON-P2, PredictSNP2, FATHMM, GWAVA, FunSeq, DANN). The output of each tool was loaded into a program that compared the observed output to the expected classification and which then calculated performance metrics such as sensitivity and specificity. The tools that we evaluated and the web addresses used can be found in Additional file 4: Table S4. We executed PROVEAN and SIFT, for which the output was reduced by retaining the following columns: “INPUT,” “PROVEAN PREDICTION (cutoff = -2.5),” and “SIFT PREDICTION (cutoff = 0.05).” For PONP-2, the output was left as is. The MSC thresholds are configurable; we downloaded the ClinVar-based thresholds for CADD 1.3 at 95% confidence interval, comparable to our method, as well as HGMD-based thresholds at 99% confidence interval, the default setting. Variants below the gene-specific thresholds were considered benign, and above the threshold pathogenic. Following the suggestion of the CADD authors, scores of variants below a threshold of 15 were considered benign, above this threshold pathogenic. We also tested CADD thresholds 20 and 25 for comparison. The output of Condel was reduced by retaining the following columns: “CHR,” “START,” “SYMBOL,” “REF,” “ALT,” “MA,” “FATHMM,” “CONDEL,” “CONDEL_LABEL.”. After running PolyPhen2, its output was reduced by retaining the positional information (“chr2:220285283|CG”) and the “prediction” column. Finally, we executed PredictSNP2, which contains the output from multiple tools. From the output VCF, we used the INFO fields “PSNPE,” “FATE,” “GWAVAE,” “DANNE,” and “FUNE” for the pathogenicity estimation outcomes according to the PredictSNP protocol for PredictSNP2 consensus, FATHMM, GWAVA, DANN, and FunSeq, respectively.

### Stratification of variants using Clinical Genomics Database

We downloaded Clinical Genomics Database (CGD; the.tsv.gz version on 1 June 2016 from http://research.nhgri.nih.gov/CGD/download/). A Java program evaluated each variant in the full set of 25,765 variants and retrieved their associate gene symbols as annotated by SnpEff. We matched the gene symbols to the genes present in CGD and retrieved the corresponding physical manifestation categories. Variants were then written out to separate files for each manifestation category (cardiovascular, craniofacial, renal, etc.). This means a variant may be output into multiple files if its gene was linked to multiple manifestation categories. However, we did prevent variants from being written out twice to the same file in the case of overlapping genes in the same manifestation categories. We output a variant into the “NotInCGD” file only if it was not located in any gene present in CGD.

### Implementation

GAVIN was implemented using Java 1.8 and MOLGENIS [32] 1.21 (http://molgenis.org). The calibration method is agnostic of the meaning of pathogenic or benign, resulting in thresholds that have balanced sensitivity and specificity. In our diagnostics practice, sensitivity is valued over specificity. We therefore adjusted the CADD and MAF thresholds to shift the balance towards sensitivity at the cost of specificity. We found a setting of 5 (adjustable in source code) achieved >90% sensitivity and this setting was used to generate final thresholds. The genome-wide classification thresholds based on CADD scores <15 for benign and >15 for pathogenic matched this high sensitivity. The full table of gene-specific thresholds used can be found at http://www.molgenis.org/gavin (for latest release) or Additional file 1: Table S1. They can be used to guide manual variant interpretation or be re-used in other tools. Source code with tool implementation details can be found at https://github.com/molgenis/gavin. All benchmarking, bootstrapping, and plotting tools can be found in this repository, as well as all data processing and calibration programs.

### Binary classification metrics

Prediction tools may classify variants as benign or pathogenic, but may also fail to reach a classification or classify a variant as VUS. Because of these three outcome states, binary classification metrics must be used with caution. We define sensitivity as the number of detected pathogenic variants (true positives) over the total number of pathogenic variants, which includes true positives, false negatives (pathogenic variants misclassified as benign), and pathogenic variants that were otherwise “missed,” i.e. classified as VUS or not classified at all. Therefore, Sensitivity = TruePositive/(TruePositive + FalseNegative + MissedPositive). We applied the same definition for specificity and define it as: Specificity = TrueNegative/(TrueNegative + FalsePositive + MissedNegative). Following this line, accuracy is then defined as (TruePositive + TrueNegative)/(TruePositive + TrueNegative + FalsePositive + FalseNegative + MissedPositive + MissedNegative).

## Additional files

Additional file 1: Table S1. GAVIN gene-specific thresholds used in the benchmark. This table can be used to look up thresholds of individual genes and allow variant interpretation by following classification rules as indicated by the column names and provided explanation. (XLSX 198 kb)
Additional file 2: Table S2. Detailed overview of all benchmark results. Each combination of tool and dataset is listed. We provide the raw counts of true positives (TP), true negatives (TN), false positives (FP), and false negatives (FN), as well as of pathogenic and benign variants that were “missed,” i.e. not correctly identified as such. From these numbers, we calculated the sensitivity and specificity. (XLSX 58 kb)
Additional file 3: Table S3. Estimate of the practical impact in clinical diagnostics of using methods of different sensitivity and specificity on a dataset with 100 benign and 10 pathogenic variants. (XLSX 50 kb)
Additional file 4: Table S4. The tools used to evaluate our benchmark variant set and the web addresses used through which they were accessed. (XLSX 51 kb)
